# Loose and Pelleted Natural Litter: Effects of Composition and Formulation on Litter Quality and Broiler Footpad Health

**DOI:** 10.3390/ani16142171

**Published:** 2026-07-13

**Authors:** Slobodan Knežević, Marko Pajić, Milica Živkov Baloš, Biljana Đurđević, Jasna Prodanov-Radulović, Zoran Ružić, Suzana Vidaković Knežević

**Affiliations:** 1 Scientific Veterinary Institute “Novi Sad”, 21000 Novi Sad, Serbia; markopajic@niv.ns.ac.rs (M.P.); milica@niv.ns.ac.rs (M.Ž.B.); biljana@niv.ns.ac.rs (B.Đ.); jasna@niv.ns.ac.rs (J.P.-R.); suzana@niv.ns.ac.rs (S.V.K.); 2 Department of Veterinary Medicine, Faculty of Agriculture, University of Novi Sad, 21000 Novi Sad, Serbia; ruzicvet@gmail.com

**Keywords:** natural bedding materials, wheat straw, wood shavings, peat, loose litter, pelleted litter, poultry litter, litter quality, footpad dermatitis, broiler welfare

## Abstract

Litter quality is a key factor influencing broiler welfare, particularly footpad health, yet the effects of loose versus pelleted natural litter formulations are not fully understood. This study assessed the impact of six treatments, composed of wheat straw, wood shavings, and peat in loose or pelleted forms, on litter moisture, pH, and footpad lesions during a 42-day fattening period. Litter condition was monitored weekly through scoring, moisture content, and pH measurements, while footpad integrity was assessed on a weekly basis throughout the fattening period. Loose litter exhibited higher moisture accumulation, greater pH variability, and earlier onset and more severe footpad lesions compared to all pelleted materials as well as wood shavings, which maintained more stable conditions and delayed lesion development. The initial physical properties and formulation of the litter materials contributed to these differences, with coarser textures potentially contributing increased mechanical stress on the footpads. Litter pH values converged and stabilized toward the end of the production cycle across all treatments. These results demonstrate that both litter composition and form significantly influence litter conditioning and broiler welfare. Careful selection and management of natural litter, with attention to moisture retention and pH stability, are essential to reduce footpad dermatitis and support optimal performance in intensive broiler production.

## Simple Summary

The quality of the bedding material used in broiler chicken housing plays an important role in keeping the birds healthy and comfortable. Poor litter can cause footpad injuries, which are painful for the chickens and can reduce their growth and production. In this study, we compared six types of natural litter, made from wheat straw, wood shavings, and peat, in either loose or pelleted forms. We measured how wet litter became, how its acidity changed, and the condition of the birds’ footpads every week during the 42-day growing period. We found that loose straw-based litter and blended litter formulation became wetter and more variable in acidity, which led to footpad injuries appearing earlier and being more severe. In contrast, pelleted litter and wood shavings were associated with better footpad health and reduced footpad damage throughout the rearing period. These findings show that both the type of material and how it is prepared affect the quality of the litter and the welfare of broilers. By choosing and managing litter carefully, farmers can reduce footpad problems, improve bird health, and support more efficient and sustainable chicken production.

## 1. Introduction

Broiler chicken meat is a source of high-quality proteins and other nutrients important for human nutrition. Additionally, the relatively low price of broiler meat has contributed to a steady increase in its consumption over the years [1]. To meet consumer demand, the poultry industry has focused on rearing fast-growing broilers that reach a body weight of approximately 3 kg within six weeks of intensive production [2].

Modern broiler production involves raising birds in enclosed and controlled farm conditions on litter. Such systems are equipped with automated feeding and watering systems, as well as environmental control systems for maintaining optimal microclimatic conditions [3]. One of the prerequisites for successful broiler production is the selection of appropriate litter material. Its importance in intensive poultry production is multifaceted. From a veterinary perspective, litter plays a crucial role in maintaining poultry health, establishing gut health, and ensuring proper zoohygienic conditions and animal welfare, while also improving production efficiency. Effective litter should have good absorbent capacity, dry quickly, resist ammonia production, be biodegradable, low in dust content, free of contaminants, non-toxic, inexpensive, and readily available [4,5,6]. Litter also acts as insulation between broilers and the floor, protecting them from cold and making their contact with a hard surface more comfortable [5].

Additionally, litter should absorb moisture resulting from drinking and defecation, dilute manure, and bind fecal material, thereby reducing direct contact between broilers and excreta. Besides having good absorbent properties, litter should retain an appropriate amount of moisture while drying, to reduce the risk of litter caking and crust formation. Finally, ideal litter should be suitable for use as a soil amendment after its use in broiler production [5].

Materials used as litter are generally by-products of crop production, forestry or other industries. Worldwide, commonly used materials include chopped straw (wheat, flax, barley), hay, chopped corn stalks, corn cobs, rice hulls, peanut shells, coconut husks, wood bark, wood chips, wood shavings, sawdust, sand, peat moss, gypsum, paper, and recycled litter, i.e., manure [5,6]. The selection of litter material depends on its local availability and cost.

In Serbia, wheat straw is the most used litter material in broiler production, either in unchopped or chopped form [7]. Among the types of straw used as litter materials, wheat, rye, oat, and barley straw are most frequently applied. Low cost, wide availability, and ease of storage represent the main advantages of straw when selecting litter material in Serbia. In addition, other materials such as wood shavings and peat are also used [8].

In addition to the choice of material, an important factor in selecting appropriate litter is its formulation. Litter can be formulated as loose or pelleted, which affects broiler comfort, thermal properties, aeration, and moisture content [9]. The preparation of pelleted litter is a complex process consisting of several stages, including material grinding, conditioning, pelleting, cooling, and storage. The production process of pelleted litter is carried out at high temperatures, making the final product microbiologically safe, but also somewhat more expensive compared to loose litter formulations.

The use of inadequate litter materials and their poor maintenance leads to increased litter moisture and deterioration of litter conditions. Litter condition directly influences the occurrence of contact dermatitis (pododermatitis), affecting areas of the skin such as the footpad, the hock region, and in more severe cases, the breast area, all of which are in constant contact with the litter [9,10].

Broilers affected by pododermatitis experience pain, which reduces their movement toward feeders and drinkers, consequently negatively affecting weight gain, feed conversion, and the overall performance of the flock [11].

Accordingly, the aim of this study was to formulate different types of litter using natural materials such as wheat straw, wood shavings, and peat, and to evaluate the effects of their composition and formulation on litter condition and the reduction in pododermatitis incidence. Special attention was given to ensuring that the applied materials are safe for broilers during the fattening period and environmentally sustainable. To achieve this aim, the study focused on assessing the conditioning state of various litter types, their impact on moisture dynamics, as well as pH as a supporting physicochemical parameter, and their influence on the occurrence of footpad dermatitis.

This research contributes to the development of practical litter formulation approaches based on combinations and physical forms of natural materials, providing insights into how natural materials can be combined and managed to improve litter quality, enhance broiler welfare, and reduce the incidence of footpad dermatitis, while maintaining environmentally sustainable production practices.

## 2. Materials and Methods

### 2.1. Housing and Diets

All procedures in this study were approved by the Ethics Committee of the Serbian Ministry of Agriculture, Forestry and Water Management (Republic of Serbia) (Approval no: 323-07-00240/2019-05). The study took place at the experimental farm of University of Novi Sad, Faculty of Agriculture in Novi Sad, Republic of Serbia.

Day-old unsexed ROSS 308 broiler chicks (*n* = 576) were randomly distributed across six treatments (*n* = 32 chicks/pen), with three replicates. Broiler chicks were raised in floor pens, with a dimension of 2.00 m^2^. The resulting stocking density of approximately 16 birds/m^2^ is consistent with commercial broiler production practices in the region, where similar densities (15–17 birds/m^2^) are commonly applied under floor-rearing systems [12,13]. Basic environmental parameters, including temperature, ventilation, and lighting, were maintained according to the requirements of the Ross hybrid [5]. Feed and water were provided ad libitum throughout the study using bell feeders and a nipple drinker line. Broiler chicks were fed a commercial diet consisting of three phases: starter (day 0 to 14), grower (day 15 to 28), and finisher (day 29 to 42).

### 2.2. Litter Management

**Figure 1:**
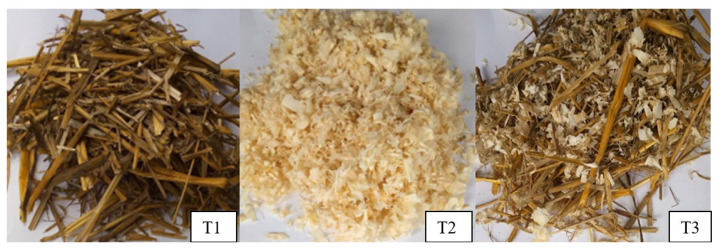
Loose litter formulations: chopped wheat straw (T1), wood shavings (T2), and a mixture of chopped wheat straw, wood shavings, and peat in equal proportions (T3).

**Figure 2:**
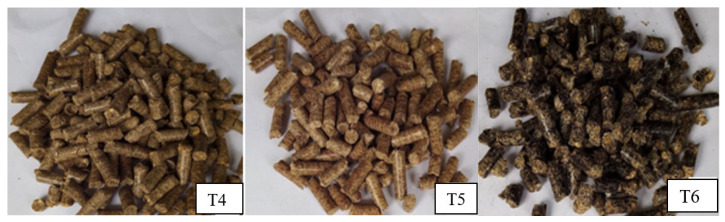
Pelleted litter formulations: pelleted wheat straw (T4), pelleted softwood (T5), and a pelleted mixture of wheat straw, wood shavings, and peat in equal proportions (T6).

By mixing different natural materials (wheat straw, wood shavings, and peat) in specific ratios, six types of litter with different compositions and formulations were produced. Three litter types were in loose form (Figure 1), including chopped wheat straw (T1), wood shavings (T2), and a mixture of chopped wheat straw, wood shavings, and peat in equal proportions (T3). Additionally, using the same materials and ratios, three more litter types were produced in pelleted form (Figure 2), including pelleted wheat straw (T4), pelleted softwood (T5), and a pelleted mixture of wheat straw, wood shavings, and peat in equal proportions (T6). The preparation of litter with different compositions and formulations included mechanical chopping of wheat straw and mechanical grinding of wheat straw, wood shavings, and peat, followed by mixing of the materials in the required proportions using a counterflow mixer. To obtain pelleted litter, all materials were previously ground and mixed according to the experimental design. The prepared raw material was then subjected to pelleting using a pellet press. The material was conveyed to the die by rollers and compressed through 6 mm die openings, after which the compacted mass was cut into pellets approximately 3 cm in length. Pelleting was performed under pressures of 140–160 bar and temperatures of up to 100 °C.

The floor pens were evenly covered with 12 kg (6 kg/m^2^) of different litter types prior to broiler chicks’ distribution, without additional litter maintenance. The initial litter depth varied according to the bulk density of each material and was approximately 6–8 cm for chopped wheat straw (5–10 cm length), 3–4 cm for wood shavings, 4–6 cm for the mixture of chopped wheat straw, wood shavings, and peat in equal proportions, and 0.8–1.2 cm for pelleted litter.

### 2.3. Litter Quality Assessment

The quality of the litter was assessed weekly on days 0, 7, 14, 21, 28, 35, and 42 according to the Welfare Quality [14] guidelines using inspection and palpation methods (by hand and foot). The litter was scored on a scale from 0 to 4, where 0 indicated completely dry and flaky litter that moves easily with the foot; 1 indicated dry litter that is not easily moved with the foot; 2 indicated litter that leaves a footprint and forms a ball when compacted but does not retain its shape well; 3 indicated litter that sticks to boots and readily forms a compact ball; and 4 indicated litter that sticks to boots once the compacted crust is broken. Scoring was performed by three experienced observers throughout the experimental period.

### 2.4. Litter Sampling, Moisture Content, and pH Measurement

Litter samples were collected weekly from all four corners and the center of each pen, including the area beneath the nipple drinker line, on days 0, 7, 14, 21, 28, 35, and 42. The subsamples collected from the four corners, the center, and the area beneath the nipple drinker line were pooled and thoroughly homogenized to form a single composite sample per pen at each sampling time point prior to laboratory analysis. Litter samples were collected across the entire litter profile, from the surface to the concrete floor, to obtain a representative estimate of the overall litter condition within each pen. The samples were packed in sampling bags and transported to the laboratory after sampling. Samples were analyzed in duplicate.

Litter moisture was measured using the gravimetric method. Samples (5 ± 0.001 g) were prepared by grinding and homogenization, and then dried in a laboratory oven (Memmert UNB 500, Schwabach, Germany) at 105 ± 2 °C until a constant weight was achieved, which required 4 h. This was followed by cooling the samples in a desiccator and measuring their mass.

The pH value of the litter was determined using the direct potentiometric method, which is based on electrochemical measurement of the pH in a water extract of the litter. Briefly, after grinding and homogenization, 10.00 ± 0.01 g of litter was weighed and mixed with 100 mL of distilled water in an extraction vessel. The mixture was shaken on a laboratory shaker for 15 min. Afterwards, the mixture was filtered, and the pH value was measured in the clear filtrate, at 20 ± 0.5 °C, using an electronic pH meter (Consort C 830, Turnhout, Belgium) calibrated with standard buffer solutions.

### 2.5. Assessment of Footpad Dermatitis

The condition of the footpad skin of the broiler chicks was evaluated by visual inspection and scoring based on the extent of the lesions. Footpad skin without visible lesions was scored as 0. Minimal damage was scored as 1 or 2, while more severe skin lesions were scored as 3 or 4 [14]. At each sampling point, all birds in each pen were individually examined for footpad dermatitis.

### 2.6. Statistical Analysis

Statistical analysis of the research results was performed using descriptive statistical indicators, as well as by testing the significance of differences (*p* < 0.05) between treatments through analysis of variance (ANOVA) and Duncan’s multiple range test. Data analysis was conducted using the statistical software R version 3.2.2 (R Foundation for Statistical Computing, Vienna, Austria). The experimental unit for all analyses was the pen. Litter quality, moisture content, and pH were analyzed at the pen level, while footpad dermatitis was summarized per pen based on individual bird scores. Statistical comparisons between treatments were performed separately for each sampling day.

## 3. Results

### 3.1. Litter Quality

The condition of the litter during broiler fattening is presented in Table 1. During the first 14 days of fattening, no statistically significant differences (*p* < 0.05) in litter condition were observed, regardless of its composition and formulation. In this period, the litter was dry and easily loosened by hand.

In the third week of fattening, i.e., on day 21, significantly better scores (*p* < 0.05) were recorded in the groups where pelletized litter was applied (T4, T5, and T6), as well as in the group with loose wood shavings (T2). During the 28-day fattening period, the assessment of litter quality ranged from 1.50 ± 0.55 (T2) to 2.33 ± 0.52 (T1), with no statistically significant differences (*p* > 0.05) between treatments. At this stage, the litter could be easily loosened by hand, although localized areas were observed where footprints remained and where the litter would clump when pressed, forming loose aggregates.

A difference (*p* < 0.05) in litter quality was observed on day 35 of the fattening period, with T1 receiving the highest score. By the end of the fattening period (day 42), no statistically significant differences (*p* > 0.05) were found between the litter compositions and formulations, with quality scores ranging from 2.33 ± 0.52 (T4 and T5) to 3.00 ± 0.63 (T6). At this stage, the litter exhibited a range of conditions, from areas where footprints remained and clumps could be formed by hand but remained loose, to areas where the litter adhered to footwear and hand-formed clumps did not disintegrate.

During the experimental period, all treatments showed a gradual deterioration in litter condition, reflected in an increase in scores.

### 3.2. Moisture Content of the Litter

The moisture content (%) of litter with different compositions and formulations during broiler fattening is presented in Table 2. At the beginning of the experiment (day 0), the moisture content ranged from 8.05 ± 0.39% in pelleted softwood (T5) to 10.60 ± 0.19% in the mixture of chopped wheat straw, wood shavings, and peat in equal proportions (T3). A slight increase in moisture was observed by day 7 of the fattening period. From day 14 until the end of the fattening period, the highest moisture values were recorded in loose litter formulations (T1, T2, and T3), while significantly (*p* < 0.05) lower values were observed in pelleted litter types (T4, T5, and T6), regardless of composition.

On days 14 and 21, the statistically (*p* < 0.05) highest moisture values were observed in T1, where chopped wheat straw was used. Towards the end of the fattening period, on day 35 and 42, the highest moisture content was recorded in T2, which consisted of loose wood shavings. From day 14 until the end of fattening period (day 42), the lowest moisture content was consistently observed in the pelleted mixture of wheat straw, wood shavings, and peat in equal proportions (T6).

At the end of the fattening period (day 42), the highest moisture content was recorded in most treatments; however, in the treatments with wheat straw (T1) and a mixture of chopped wheat straw, wood shavings, and peat in equal proportions (T3), the highest moisture content was observed on day 28 of the fattening period.

### 3.3. Litter pH Value

The pH values of litter during broiler fattening are presented in Table 3. Initial pH varied among litter materials, ranging from acidic values in wood shavings (T2), pelleted softwood (T5), and pelleted mixtures (T6) to alkaline values in wheat straw-based litter (T1, T3, and T4).

During the fattening period, pH generally increased and remained within a slightly alkaline range for all treatments. Although some statistically significant differences were observed among litter types at individual sampling points (*p* < 0.05), these differences decreased over time, and no significant differences were detected at day 42 (*p* > 0.05).

### 3.4. Footpad Dermatitis

Table 4 presents the footpad condition of broilers during the fattening period. Footpad skin in broilers reared on pelleted litter (T4, T5, and T6) remained intact until day 21 of fattening, when the first signs of lesions began to appear, and until day 28 in broilers reared on wood shavings (T2). The earliest signs of footpad lesions were observed as early as day 7 in broilers raised on chopped wheat straw (T1) and on the loose mixture of chopped wheat straw, wood shavings, and peat in equal proportions (T3). By the end of the fattening period, these treatments exhibited the highest degree of footpad damage.

Up to day 21 of fattening, broilers reared on wood shavings (T2) and pelleted litter formulations showed statistically significantly (*p* < 0.05) lower footpad lesion scores, indicating better footpad condition compared to other treatments. This trend persisted until the end of the fattening period. On day 42, the lowest degree of footpad lesions was observed in treatments T2, T4, and T6, where loose wood shavings and pelleted litter formulations were used with the differences being statistically significant (*p* < 0.05). The highest degree of footpad lesions was recorded in broilers from treatment T3, where a loose mixture of chopped wheat straw, wood shavings, and peat in equal proportions was applied.

## 4. Discussion

In recent decades, with the increase in broiler production, there has been a growing number of studies focused on identifying alternative materials for litter formulation in broiler housing. For a material to qualify as suitable litter, it must be readily available, inexpensive, highly absorbent, capable of drying quickly, free from dust and contaminants, have low thermal conductivity, positively influence broiler welfare and performance, and be safe for the health of the birds [5,6].

Broilers may consume a portion of the litter, which is estimated to account for approximately 6% of their total feed intake [6]. For this reason, natural materials, such as wheat straw, wood shavings, and peat, are preferred for use as broiler litter. Wheat straw is an agricultural biomass collected immediately after wheat harvest in the summer months (June or July). The yield of straw is equivalent to the yield of the crop itself (1:1 ratio), and due to its availability and quantity, it is frequently used in Serbia, either chopped or unchopped, as litter material for various animal species [15], including broilers.

The main attributes of wood shavings as broiler litter are ease of handling, the potential for reuse, and good absorbent capacity [16]. Peat is considered suitable as litter due to its ability to quickly absorb and release excess moisture [8].

Coarse and rough materials may cause abrasive effects on broiler body parts such as legs and breast, which can reduce carcass quality [6,17]. To prevent such issues in the present study, wheat straw was machine-chopped to lengths of 5–10 cm, and wood shavings were sourced from softwood species. The selected straw particle size was chosen to reflect common commercial practice in the region, where mechanically chopped straw of similar length is frequently used in floor-based broiler production systems.

The amount of litter applied in broiler houses varies depending on management practices, litter type, and housing conditions. Commercial recommendations generally suggest an initial litter depth of 2–4 cm [5]. However, scientific studies frequently employ greater litter quantities, such as 3 kg/m^2^, 6 kg/m^2^ [18], or even litter depths of 15 cm, which is approximately equivalent to 8 kg/m^2^ [19], depending on the litter material, climatic conditions, production system, and housing design [20]. In the present study, a litter application rate of 6 kg/m^2^ was used to ensure adequate floor coverage and moisture absorption throughout the rearing period, reflecting both experimental and commercial practice.

Conversely, dust and fine particles from excessively dry materials can adversely affect the respiratory tract of broilers, leading to respiratory problems [6]. To prevent this, natural materials and their combinations were pelleted into cylindrical shapes (3 cm in length and 6 mm in diameter), which contain less dust and fine particles. Pelleting also increases the density of the materials: while the density of raw materials is approximately 200 kg/m^3^, pellet density ranges between 770 and 790 kg/m^3^ [21], facilitating storage and handling.

As the fattening period progressed, the litter became increasingly wet due to normal water spillage and excretion, leading to a decline in its condition. At the end of the fattening period, litter quality was evaluated with scores above 2.33 ± 0.52. During this stage, the litter occasionally adhered to footwear, and hand-formed clumps did not disintegrate, indicating high moisture content. This can be explained by the saturation of the litter with moisture, primarily resulting from spilled drinking water and excreta [22], in combination with limited moisture removal through evaporation and ventilation, which together determine the overall litter moisture balance.

The litter scoring system was useful for monitoring overall changes in litter condition throughout the production cycle. However, despite clear differences in footpad dermatitis severity among treatments, litter quality scores did not differ significantly at the end of the rearing period. This apparent discrepancy may reflect the limited sensitivity of visual scoring methods to detect subtle differences in litter characteristics, particularly at the surface layer where birds are in direct contact with the litter. Furthermore, litter scores provide a generalized assessment of litter condition and may not fully reflect localized variations within the pen. Therefore, although litter scoring represents a practical tool for evaluating overall litter quality, it may be less effective in identifying conditions most closely associated with the development of footpad dermatitis.

The initial moisture content of the litter in this study ranged from 8.05 ± 0.39% (T5) to 10.60 ± 0.19% (T3), which is comparable to previous reports [22,23]. Differences in initial moisture content can be attributed to the specific characteristics of the materials used and their inherent water content [24]. Similar observations were reported by Brink et al. [23], who found moisture levels of approximately 4% in wood shavings, flax, chopped wheat straw, and broken flax pellets, whereas higher values were observed for peat (18.06%) and corn silage (35.69%). However, these differences are expected to equilibrate with ambient conditions during use [25]. Therefore, moisture dynamics during rearing are more relevant indicators of litter performance and their effects on broiler welfare than the small differences observed before bird placement.

By the end of the fattening period, an increase in moisture content was observed in all litter types, exceeding fivefold, due to the accumulation of excreta, spilled drinking water, and air relative humidity [26]. While higher litter moisture may reduce dust emissions, it can also promote the release of unpleasant odors, increase ammonia levels in the housing environment [27], and contribute to the occurrence of health issues such as footpad dermatitis [28,29,30]. Because water spillage and excreta deposition were not quantified separately in the present study, their individual contributions to litter moisture accumulation could not be distinguished. However, all treatment groups were maintained under identical housing, management, and environmental conditions; therefore, the observed differences in litter moisture were most likely associated with the moisture absorption, retention, and drying characteristics of the litter materials themselves.

Although the overall trend was an increase in litter moisture throughout the production cycle, temporary reductions were observed in some treatments between days 28 and 35. Such fluctuations may be related to differences in the physical properties of the bedding materials, including particle size distribution, porosity, moisture retention capacity, and drying dynamics. Moisture distribution within broiler houses is also spatially heterogeneous, with wetter areas typically occurring near drinker lines and along house walls, while central areas tend to remain drier. Variations in ventilation efficiency and evaporative water loss may also contribute to short-term changes in litter moisture, even in the absence of litter replacement or other mechanical interventions [31]. Interestingly, loose wood shavings treatment (T2) exhibited relatively higher litter moisture content towards the end of the trial, while simultaneously maintaining low footpad dermatitis scores. This suggests that litter physical characteristics, rather than moisture content alone, play a significant role in determining footpad health. In particular, the structural properties of loose wood shavings may have provided a cushioning effect and may have reduced the effective contact between the footpads and moisture-saturated litter due to their improved drainage and aeration characteristics.

Maintaining litter in an appropriate moisture condition is an important aspect of broiler production. Wet litter has been associated with an increased risk to bird health and negative effects on welfare [11,32], whereas excessively dry litter can cause problems related to dust, including respiratory issues and reduced weight gain [33]. Consequently, proper litter management, including the selection of suitable bedding materials and effective environmental control, particularly adequate ventilation, is essential for maintaining optimal litter quality and supporting bird welfare and production performance.

The observed increase in litter pH during the fattening period can be attributed to the accumulation of excreta and associated biochemical processes, particularly the microbial degradation of uric acid into urea and ammonia [20,24,34]. These processes contribute to a gradual shift toward alkaline conditions, regardless of the initial differences between litter types. The initially lower pH values observed in wood-based and peat-containing materials are consistent with previous findings, as such materials typically exhibit acidic properties [20]. However, this initial advantage diminishes over time due to the increasing influence of metabolic waste.

The convergence of pH values among treatments toward the end of the fattening period suggests that litter composition and formulation have a limited long-term effect on pH dynamics under intensive production conditions. Although statistical differences among treatments were detected at certain sampling points, the magnitude of these differences was generally small, particularly during the later stages of the production cycle when pH values converted across treatments. This suggests that the practical significance of treatment-related differences in pH was limited compared with the influence of manure accumulation and environmental conditions. In contrast to pH, moisture content remained more consistently associated with differences in footpad health. These findings suggest that moisture was the more influential environmental factor affecting footpad health under the conditions of the present study. As reported in a previous study, factors such as excreta accumulation, water management, and microclimatic conditions are considered important determinants of the litter environment and may have had a stronger influence on litter pH during the production cycle than the initial litter composition [22].

From a practical perspective, the shift toward slightly alkaline conditions may be relevant because higher litter pH has been associated with increased ammonia volatilization. Since ammonia emissions were not measured in the present study, no direct conclusions can be drawn regarding ammonia release. Nevertheless, litter moisture and pH are widely recognized as important indirect indicators of litter conditions that may favor ammonia formation and volatilization in poultry housing systems. Maintaining litter pH below 7.0 has been recommended as a management strategy to reduce the risk of ammonia emissions and support air quality in broiler houses [28].

It should also be noted that pH conditions within litter are not uniform and may vary considerably with depth and moisture content. Previous study has shown that caked surface layers may exhibit lower pH values, whereas more alkaline conditions can occur in deeper litter layers due to ongoing microbial activity and ammonia generation [35]. Because litter samples in the present study were collected across the full litter depth and homogenized prior to analysis, the measured pH values represent the overall litter environment rather than the specific surface conditions directly experienced by broilers. Therefore, although different litter materials exhibited distinct initial pH characteristics, manure accumulation, moisture conditions, and other environmental factors appeared to have a greater influence on litter pH during the production cycle.

Footpad condition is a critical indicator of broiler health and welfare, and it also has important implications for economic efficiency and food safety [9,36,37].

In the present study, the first signs of footpad dermatitis were observed as early as day 7 of fattening in broilers reared on loose litter formulations, with the exception of broilers raised on wood shavings, which showed delayed lesion development. The appearance of footpad lesions in broilers reared on pelleted litter was recorded later, around day 21 of fattening. Visual observations indicated that pelleted litter began to show signs of disintegration from day 28 onwards, with progressive breakdown towards the end of the rearing period. However, the rate of pellet disintegration and associated changes in litter depth were not quantified in this study and warrant further investigation. By the end of the fattening period, broilers reared on loose litter formulations exhibited a significantly higher degree of footpad lesions, except in the case of wood shavings.

The early appearance of footpad dermatitis (day 7) observed in the present study is consistent with previous reports indicating that footpad dermatitis can develop within the first week of life under commercial broiler conditions [38]. The occurrence of lesions at this early stage, despite relatively low mean litter moisture values, suggests that lesion initiation may be influenced by factors beyond average litter moisture alone. By contrast, the delayed onset of lesions in broilers reared on pelleted litter indicates that the physical structure of the bedding may have initially provided a more favorable contact surface for the birds. The observed temporal relationship between litter deterioration and increasing footpad lesion severity further supports the importance of litter condition in the development of footpad dermatitis. In addition, the coarser structure of loose litter materials may have contributed to the earlier development of lesions through increased mechanical irritation of the footpad surface. However, as the mechanical effects of litter texture were not directly evaluated in the present study, this explanation should be considered a potential contributing factor rather than a confirmed mechanism. In pelleted litter, the initial structural integrity may have reduced immediate contact between excreta and the bird-occupied surface; however, progressive disintegration likely diminished this advantage over time. The lower incidence of footpad lesions on pelleted litter compared to loose formulations has also been reported previously [39]. However, direct comparison of the results should be interpreted with caution due to differences in experimental design among studies, particularly with regard to litter depth and the extent to which pelleted materials expand and change their physical characteristics after placement. The size and coarseness of materials used in loose litter formulations may have contributed to the damage observed on the footpad skin of broilers. Similar results were reported by Žikić et al. [40] and Đukić Stojčić et al. [7] in studies comparing chopped and unchopped straw as litter.

Some studies indicate a lower incidence of footpad lesions in broilers raised on wood shavings compared to straw [36,41], which is consistent with the findings of the present study. These observations highlight the importance of litter type and physical properties in minimizing footpad damage and maintaining broiler welfare.

Overall, the findings of this study demonstrate that the physical and chemical properties of litter, including its formulation, moisture content, and pH, play an important role in broiler welfare, particularly in relation to footpad health. Loose, coarse materials tended to retain more moisture and exhibited greater variability in pH. This increased moisture retention is likely associated with surface sealing and reduced evaporation rates, which can limit water vapour removal from the litter surface [42]. Together with the physical texture of these materials, such conditions may have contributed to the earlier onset and greater severity of footpad lesions. However, the results also indicate that moisture alone does not fully explain footpad health outcomes, as the physical characteristics of the litter material likely influenced lesion development. Furthermore, litter surface condition, as assessed through litter quality scoring, appeared to be closely associated with footpad lesion development and may represent a more relevant indicator of footpad health risk than bulk litter moisture content. In contrast, pelleted litter and wood shavings were associated with more stable conditions, delayed lesion onset and better footpad integrity throughout the fattening period, suggesting that both moisture-related and structural properties of litter contribute to footpad health, while the specific role of pH appears to be secondary under the conditions of this study.

These results emphasize that both the choice of litter material and its formulation directly influence litter conditioning throughout the fattening period, affecting moisture retention, pH dynamics, and the mechanical impact on broilers. Effective litter management, therefore, requires careful consideration of material characteristics and formulation, together with active management conditions such as ventilation, air movement, heating, and water distribution, in order to maintain optimal litter quality and reduce the risk of footpad dermatitis.

Future research could focus on evaluating additional litter formulations and combinations of natural materials to further optimize moisture retention and pH stability, thereby minimizing footpad dermatitis. Studies under varying environmental and management conditions could also provide insight into the long-term effects of litter properties on broiler performance, health, and housing air quality, offering practical guidance for large-scale production systems.

## 5. Conclusions

This study demonstrates that the composition and formulation of litter have a direct impact on broiler welfare, particularly on footpad health. Loose, coarse materials tended to retain more moisture and exhibit greater pH variability, which may have contributed to earlier and more severe footpad lesions. In contrast, pelleted litter and wood shavings were associated with better footpad integrity and delayed lesion onset. These effects were likely influenced not only by moisture conditions but also by the physical characteristics of litter materials, including their structure and surface properties.

The findings highlight the importance of litter form as a primary determinant of footpad health under the conditions of this study, rather than general statements about balancing moisture content and monitoring litter pH. Under the conditions of the present study, moisture content appeared to be more strongly associated with footpad health than pH, particularly at the litter–foot contact surface where lesions develop.

From a practical perspective, both wood shavings and pelleted litter were associated with lower overall footpad lesion severity compared with loose straw-based litter, although responses varied among pelleted formulations over time. Pelleted litter was associated with a delayed onset of footpad lesions and lower severity in the early stages; however, this early advantage was not consistently maintained across all pelleted treatments. By the end of the fattening period, two of the pelleted litter formulations showed lesion severity comparable to that observed in broilers reared on wood shavings, whereas differences among treatments were less pronounced than in the early stages. Loose straw-based litter was associated with the highest risk of early lesion development and greater overall severity.

These results suggest that poultry producers may benefit from selecting litter materials that maintain structural integrity and surface dryness throughout the production cycle. Pelleted systems may be advantageous only when pellet disintegration is delayed or effectively managed, whereas loose straw-based litter may require more intensive management to reduce the formation of persistently wet surface areas.

Appropriate litter management, including careful material selection and formulation, is essential for minimizing health risks, improving broiler welfare, and supporting optimal production performance.

## Figures and Tables

**Table 1:** Litter condition during broiler fattening.

Treatments	Days						
	0	7	14	21	28	35	42
T1	0.00 ± 0.00 ^D^	0.00 ± 0.00 ^D^	0.17 ± 0.41 ^D^	1.67 ± 0.52 ^aC^	2.33 ± 0.52 ^B^	2.67 ± 0.52 ^aAB^	2.83 ± 0.41 ^A^
T2	0.00 ± 0.00 ^C^	0.00 ± 0.00 ^C^	0.17 ± 0.41 ^C^	0.50 ± 0.55 ^bC^	1.50 ± 0.55 ^B^	1.83 ± 0.41 ^bAB^	2.33 ± 0.82 ^A^
T3	0.00 ± 0.00 ^D^	0.00 ± 0.00 ^D^	0.17 ± 0.41 ^D^	1.50 ± 0.55 ^aC^	2.17 ± 0.75 ^B^	2.50 ± 0.55 ^abAB^	2.83 ± 0.41 ^A^
T4	0.00 ± 0.00 ^C^	0.00 ± 0.00 ^C^	0.00 ± 0.00 ^C^	0.17 ± 0.41 ^bC^	1.67 ± 0.82 ^B^	2.00 ± 0.63 ^abAB^	2.33 ± 0.52 ^A^
T5	0.00 ± 0.00 ^C^	0.00 ± 0.00 ^C^	0.00 ± 0.00 ^C^	0.17 ± 0.41 ^bC^	1.50 ± 0.84 ^B^	1.83 ± 0.75 ^bAB^	2.33 ± 0.52 ^A^
T6	0.00 ± 0.00 ^C^	0.00 ± 0.00 ^C^	0.00 ± 0.00 ^C^	0.50 ± 0.55 ^bC^	1.83 ± 0.75 ^B^	2.50 ± 0.55 ^abA^	3.00 ± 0.63 ^A^

Values are expressed as mean ± standard deviation (*n* = 3). Values within the same column marked with different lowercase superscript letters (a, b) indicate statistically significant differences (*p* < 0.05) among litter types. Values within the same row marked with different uppercase superscript letters (A, B, C, D) indicate statistically significant differences (*p* < 0.05) over the duration of the broiler fattening period within the treatment. Treatments: T1—chopped wheat straw; T2—wood shavings; T3—mixture of chopped wheat straw, wood shavings, and peat (1:1:1); T4—pelleted wheat straw; T5—pelleted softwood; T6—pelleted mixture of wheat straw, wood shavings, and peat (1:1:1).

**Table 2:** Moisture content (%) of litter during broiler fattening.

Treatments	Days						
	0	7	14	21	28	35	42
T1	8.60 ± 0.89 ^dF^	12.71 ± 0.15 ^cE^	48.94 ± 0.28 ^aD^	51.35 ± 0.47 ^aC^	56.53 ± 0.42 ^bA^	49.39 ± 0.40 ^cD^	53.95 ± 0.30 ^cB^
T2	9.43 ± 0.26 ^bcG^	11.77 ± 0.53 ^dF^	32.34 ± 0.82 ^cE^	42.96 ± 0.45 ^cD^	49.67 ± 0.26 ^cC^	54.97 ± 0.26 ^aB^	57.54 ± 0.40 ^aA^
T3	10.60 ± 0.19 ^aG^	15.12 ± 0.23 ^bF^	38.80 ± 0.26 ^bE^	45.91 ± 0.57 ^bD^	57.25 ± 0.44 ^aA^	53.91 ± 0.19 ^bC^	55.34 ± 0.45 ^bB^
T4	8.83 ± 0.08 ^cdF^	19.69 ± 0.51 ^aE^	29.28 ± 0.54 ^dD^	38.72 ± 0.52 ^dC^	41.43 ± 0.34 ^eB^	47.33 ± 0.28 ^dA^	47.78 ± 0.25 ^dA^
T5	8.05 ± 0.39 ^dG^	14.55 ± 0.56 ^bF^	25.22 ± 0.41 ^eE^	37.48 ± 0.41 ^eD^	42.55 ± 0.26 ^dC^	44.51 ± 0.46 ^eB^	47.63 ± 0.28 ^dA^
T6	9.92 ± 0.25 ^abG^	12.10 ± 0.15 ^cdF^	21.70 ± 0.27 ^fE^	28.74 ± 0.55 ^fD^	40.94 ± 0.38 ^eB^	38.91 ± 0.26 ^fC^	44.09 ± 0.24 ^eA^

Values are expressed as mean ± standard deviation (*n* = 3). Values within the same column marked with different lowercase superscript letters (a, b, c, d, e, f) indicate statistically significant differences (*p* < 0.05) among litter types. Values within the same row marked with different uppercase superscript letters (A, B, C, D, E, F, G) indicate statistically significant differences (*p* < 0.05) over the duration of the broiler fattening period within the treatment. Treatments: T1—chopped wheat straw; T2—wood shavings; T3—mixture of chopped wheat straw, wood shavings, and peat (1:1:1); T4—pelleted wheat straw; T5—pelleted softwood; T6—pelleted mixture of wheat straw, wood shavings, and peat (1:1:1).

**Table 3:** pH values of litter during broiler fattening.

Treatments	Days						
	0	7	14	21	28	35	42
T1	8.1 ± 0.1 ^aABC^	7.0 ± 0.5 ^abE^	7.3 ± 0.3 ^bcDE^	8.5 ± 0.1 ^aA^	8.1 ± 0.2 ^aAB^	7.9 ± 0.1 ^aBC^	7.6 ± 0.3 ^CD^
T2	6.1 ± 0.3 ^eC^	6.9 ± 0.5 ^abB^	7.3 ± 0.7 ^bcAB^	7.9 ± 0.2 ^abA^	7.5 ± 0.5 ^abcAB^	7.3 ± 0.2 ^abcAB^	7.9 ± 0.3 ^A^
T3	7.5 ± 0.2 ^cCD^	7.3 ± 0.1 ^aD^	8.2 ± 0.3 ^aAB^	8.3 ± 0.1 ^aA^	7.9 ± 0.2 ^abAB^	7.9 ± 0.2 ^abABC^	7.8 ± 0.1 ^BC^
T4	7.8 ± 0.1 ^bA^	7.0 ± 0.3 ^abB^	7.5 ± 0.2 ^bAB^	7.8 ± 0.6 ^abA^	7.1 ± 0.2 ^cB^	7.3 ± 0.6 ^abcAB^	7.9 ± 0.2 ^A^
T5	5.7 ± 0.1 ^fC^	7.0 ± 0.2 ^abAB^	6.7 ± 0.1 ^bcB^	7.4 ± 0.9 ^bAB^	7.1 ± 0.6 ^cAB^	7.2 ± 0.4 ^bcAB^	7.8 ± 0.2 ^A^
T6	6.8 ± 0.0 ^dCD^	6.5 ± 0.3 ^bD^	6.7 ± 0.2 ^cCD^	7.8 ± 0.2 ^abA^	7.4 ± 0.1 ^bcB^	7.0 ± 0.3 ^cC^	7.8 ± 0.2 ^A^

Values are expressed as mean ± standard deviation (*n* = 3). Values within the same column marked with different lowercase superscript letters (a, b, c, d, e, f) indicate statistically significant differences (*p* < 0.05) among litter types. Values within the same row marked with different uppercase superscript letters (A, B, C, D, E) indicate statistically significant differences (*p* < 0.05) over the duration of the broiler fattening period within the treatment. Treatments: T1—chopped wheat straw; T2—wood shavings; T3—mixture of chopped wheat straw, wood shavings, and peat (1:1:1); T4—pelleted wheat straw; T5—pelleted softwood; T6—pelleted mixture of wheat straw, wood shavings, and peat (1:1:1).

**Table 4:** Footpad condition in broilers during fattening.

Treatments	Days					
	7	14	21	28	35	42
T1	0.20 ± 0.40 ^aD^	0.24 ± 0.43 ^aD^	1.04 ± 0.75 ^aC^	1.39 ± 0.75 ^aB^	1.65 ± 0.86 ^aA^	1.67 ± 0.95 ^bA^
T2	0.00 ± 0.00 ^cC^	0.00 ± 0.00 ^bC^	0.00 ± 0.00 ^bC^	0.13 ± 0.33 ^dB^	0.23 ± 0.47 ^cA^	0.31 ± 0.58 ^dA^
T3	0.10 ± 0.31 ^bD^	0.22 ± 0.42 ^aD^	0.92 ± 0.78 ^aC^	1.45 ± 0.72 ^aB^	1.84 ± 0.89 ^aA^	1.91 ± 0.88 ^aA^
T4	0.00 ± 0.00 ^cB^	0.00 ± 0.00 ^bB^	0.06 ± 0.24 ^bB^	0.35 ± 0.56 ^cA^	0.36 ± 0.58 ^cA^	0.40 ± 0.67 ^dA^
T5	0.00 ± 0.00 ^cC^	0.00 ± 0.00 ^bC^	0.01 ± 0.10 ^bC^	0.56 ± 0.71 ^bB^	0.74 ± 1.02 ^bAB^	0.81 ± 1.04 ^cA^
T6	0.00 ± 0.00 ^cC^	0.00 ± 0.00 ^bC^	0.03 ± 0.17 ^bC^	0.07 ± 0.26 ^dC^	0.17 ± 0.37 ^cB^	0.29 ± 0.54 ^dA^

Values are expressed as mean ± standard deviation (*n* = 3). Values within the same column marked with different lowercase superscript letters (a, b, c, d) indicate statistically significant differences (*p* < 0.05) among litter types. Values within the same row marked with different uppercase superscript letters (A, B, C, D) indicate statistically significant differences (*p* < 0.05) over the duration of the broiler fattening period within the treatment. Treatments: T1—chopped wheat straw; T2—wood shavings; T3—mixture of chopped wheat straw, wood shavings, and peat (1:1:1); T4—pelleted wheat straw; T5—pelleted softwood; T6—pelleted mixture of wheat straw, wood shavings, and peat (1:1:1).

## Data Availability

The original contributions presented in the study are included in the article, further inquiries can be directed to the corresponding author.

## References

[1] Kralik G., Kralik Z., Grčević M., Hanžek D. (2018). Quality of chicken meat. Anim. Husb. Nutr..

[2] Aviagen (2022). Ross 308/308 FF Broiler: Performance Objectives. https://aviagen.com/assets/Tech_Center/Ross_Broiler/RossxRoss308-BroilerPerformanceObjectives2022-EN.pdf.

[3] FAO (2016). Greenhouse gas emissions and fossil energy use from poultry supply chains: Guidelines for assessment. Livestock Environmental Assessment and Performance Partnership.

[4] Lacy M.P., Bell D., Weaver W. (2002). Broiler management. Commercial Chicken Meat and Egg Production.

[5] Aviagen (2025). Broiler Management Handbook. https://aviagen.com/assets/Tech_Center/Ross_Broiler/Aviagen-ROSS-Broiler-Handbook-EN.pdf.

[6] Diarra S., Lameta S., Amosa F., Anand S. (2021). Alternative bedding materials for poultry: Availability, efficacy, and major constraints. Front. Vet. Sci..

[7] Đukić Stojčić M., Bjedov S., Žikić D., Perić L., Milošević N. (2016). Effect of straw size and microbial amendment of litter on certain litter quality parameters, ammonia emission, and footpad dermatitis in broilers. Arch. Anim. Breed..

[8] Živkov Baloš M., Knežević S., Pajić M., Popov N., Jakšić S., Vidaković Knežević S., Mihaljev Ž., Bugarski D. (2020). The effects of bedding material containing peat moss on broiler production performance and fertilizing value of the litter. Arch. Vet. Med..

[9] Shepherd E.M., Fairchild B.D. (2010). Footpad dermatitis in poultry. Poult. Sci..

[10] Koshchaev I., Mezinova K., Ryadinskaya A., Sorokina N., Chuev S. (2020). Identification of cases of pododermatitis in broiler chickens when feeding a probiotic feed additive. E3S Web Conf. EDP Sci..

[11] de Jong I.C., Gunnink H., Van Harn J. (2014). Wet litter not only induces footpad dermatitis but also reduces overall welfare, technical performance, and carcass yield in broiler chickens. J. Appl. Poult. Res..

[12] Mitrović S., Škrbić Z., Bogosavljević-Bošković S., Ostojić D., Djermanović V. (2005). Effect of housing density, duration of fattening and initial body mass of one day old chickens on production of broiler meat of Cobb hybrid. Biotechnol. Anim. Husb..

[13] Mitrović S., Đermanović V., Radivojević M., Rajić Z., Živković D., Ostojić Đ., Filipović N. (2010). The influence of population density and duration of breeding on broiler chickens productivity and profitability. Afr. J. Biotechnol..

[14] Welfare Quality (2009). Welfare Quality Assessment Protocol for Poultry. https://www.welfarequalitynetwork.net/media/1293/poultry-protocol-watermark-6-2-2020.pdf.

[15] Jovanović ǈ., Supić D., Đuričković A. (2021). Available Potential of Harvest Residues for Bioenergy Production in the Territory of Municipalities: Bečej, Srbobran, Kula and Vrbas.

[16] Garcia R.G., Almeida Paz I.C.L., Caldara F.R., Nääs I.A., Pereira D.F., Ferreira V.M.O.S. (2012). Selecting the most adequate bedding material for broiler production in Brazil. Rev. Bras. Cienc. Avic..

[17] Almeida Paz I.C.L., Garcia R., Bernardi R., Nääs I., Caldara F.R., Freitas L.W., Seno L.O., Ferreira V.M.O.S., Pereira D.F., Cavichiolo F. (2010). Selecting appropriate bedding to reduce locomotion problems in broilers. Rev. Bras. Cienc. Avic..

[18] Fort G., Yeter B. (2021). Use of Gyttja as litter material in broiler houses. Tarim Doga Derg..

[19] Petek M., Üstüner H., Yeşilbağ D. (2014). Effects of stocking density and litter type on litter quality and growth performance of broiler chicken. Kafkas Üniv. Vet. Fak. Derg..

[20] Munir M.T., Belloncle C., Irle M., Federighi M. (2019). Wood-based litter in poultry production: A review. Worlds Poult. Sci. J..

[21] McMullen J., Fasina O.O., Wood C.W., Feng Y. (2005). Storage and handling characteristics of pellets from poultry litter. Appl. Eng. Agric..

[22] Dunlop M.W., McAuley J., Blackall P.J., Stuetz R.M. (2016). Water activity of poultry litter: Relationship to moisture content during a grow-out. J. Environ. Manag..

[23] Brink M., Janssens G.P.J., Demeyer P., Bağci Ö., Delezie E. (2022). Ammonia concentrations, litter quality, performance and some welfare parameters of broilers kept on different bedding materials. Br. Poult. Sci..

[24] Abougabal M.S., Taboosha M.F. (2023). Effect of different types of available litter materials on the performance and welfare of broiler chickens. Azhar J. Agric. Res..

[25] Dunlop M.W., Blackall P.J., Stuetz R.M. (2016). Odour emissions from poultry litter–A review litter properties, odour formation and odorant emissions from porous materials. J. Environ. Manag..

[26] Garcês A.P.J.T., Afonso S.M.S., Chilundo A., Jairoce C.T.S. (2013). Evaluation of different litter materials for broiler production in a hot and humid environment: 1. Litter characteristics and quality. J. Appl. Poult. Res..

[27] Knežević S., Vidaković Knežević S., Pajić M., Ružić Z., Stojčić M.Đ., Živkov-Baloš M., Đorđević M. (2021). Influence of different litter types on ammonia and carbon dioxide emission in broiler production. Eur. Poult. Sci..

[28] Ashworth A.J., Chastain J.P., Moore P.A. (2020). Nutrient characteristics of poultry manure and litter. Anim. Manure Prod. Charact. Environ. Concerns Manag..

[29] Pal A., Bailey M.A., Talorico A.A., Krehling J.T., Macklin K.S., Price S.B., Buhr R.J., Bourassa D.V. (2021). Impact of poultry litter Salmonella levels and moisture on transfer of Salmonella through associated in vitro generated dust. Poult. Sci..

[30] Mou C.T., Czarick M., Fairchild B.D. (2025). Evaluation of the effects of maintaining a moderate humidity (50–60%) and increased air movement on litter moisture and footpad health in a commercial broiler house. J. Appl. Poult. Res..

[31] Brink M., Janssens G.P., Delezie E. (2022). How do moisture content, friability, and crust development of litter influence ammonia concentrations in broiler production?. Livest. Sci..

[32] Strašifták J., Juhás P. (2023). The effect of a bedding materials on performance, welfare and behavior of broiler chickens: A review. J. Cent. Eur. Agric..

[33] Lai H.T., Nieuwland M.G., Kemp B., Aarnink A.J., Parmentier H.K. (2009). Effects of dust and airborne dust components on antibody responses, body weight gain, and heart morphology of broilers. Poult. Sci..

[34] Rogeri D.A., Ernani P.R., Mantovani A., Lourenço K.S. (2016). Composition of poultry litter in Southern Brazil. Rev. Bras. Cienc. Solo.

[35] Miles D.M., Rowe D.E., Owens P.R. (2008). Winter broiler litter gases and nitrogen compounds: Temporal and spatial trends. Atmos. Environ..

[36] Boussaada T., Lakhdari K., Meradi S.A.B.S. (2022). Effects of common litter types and their physicochemical properties on the welfare of broilers. Vet. World.

[37] Alabi O.M., Olagunju S.O., Aderemi F.A., Lawal T.E., Oguntunji A.O., Ayoola M.O., Oladejo O.A., Adeleye B.E., Adewumi A.A., Alabi B.D. (2024). Effect of litter management systems on incidence and severity of footpad dermatitis among broilers at finisher stage. Transl. Anim. Sci..

[38] Hashimoto S., Yamazaki K., Obi T., Takase K. (2011). Footpad dermatitis in broiler chickens in Japan. J. Vet. Med. Sci..

[39] Kheravii S.K., Swick R.A., Choct M., Wu S.B. (2017). Potential of pelleted wheat straw as an alternative bedding material for broilers. Poult. Sci..

[40] Zikic D., Djukic-Stojcic M., Bjedov S., Peric L., Stojanovic S., Uscebrka G. (2017). Effect of litter on development and severity of foot-pad dermatitis and behavior of broiler chickens. Rev. Bras. Cienc. Avic..

[41] Sirri F., Minelli G., Folegatti E., Lolli S., Meluzzi A. (2007). Foot dermatitis and productive traits in broiler chickens kept with different stocking densities, litter types and light regimen. Ital. J. Anim. Sci..

[42] Dunlop M.W., Blackall P.J., Stuetz R.M. (2015). Water addition, evaporation and water holding capacity of poultry litter. Sci. Total Environ..

